# Validation of Global Diet Quality Score Among Nonpregnant Women of Reproductive Age in India: Findings from the Andhra Pradesh Children and Parents Study (APCAPS) and the Indian Migration Study (IMS)

**DOI:** 10.1093/jn/nxab217

**Published:** 2021-10-23

**Authors:** Mika Matsuzaki, Nick Birk, Sabri Bromage, Liza Bowen, Carolina Batis, Teresa T Fung, Yanping Li, Meir J Stampfer, Megan Deitchler, Walter C Willett, Wafaie W Fawzi, Sanjay Kinra, Shilpa N Bhupathiraju

**Affiliations:** Johns Hopkins Bloomberg School of Public Health, Baltimore, MD, USA; Harvard T.H. Chan School of Public Health, Boston, MA, USA; Harvard T.H. Chan School of Public Health, Boston, MA, USA; King's College, Department of Population Health Sciences, London, United Kingdom; CONACYT—Health and Nutrition Research Center, National Institute of Public Health, Cuernavaca, Mexico; Harvard T.H. Chan School of Public Health, Boston, MA, USA; Department of Nutrition, Simmons University, Boston, MA, USA; Harvard T.H. Chan School of Public Health, Boston, MA, USA; Harvard T.H. Chan School of Public Health, Boston, MA, USA; Channing Division of Network Medicine, Brigham and Women's Hospital, Boston, MA, USA; Intake–Center for Dietary Assessment, FHI Solutions, Washington, DC, USA; Harvard T.H. Chan School of Public Health, Boston, MA, USA; Channing Division of Network Medicine, Brigham and Women's Hospital, Boston, MA, USA; Harvard T.H. Chan School of Public Health, Boston, MA, USA; London School of Hygiene and Tropical Medicine, London, United Kingdom; Harvard T.H. Chan School of Public Health, Boston, MA, USA; Channing Division of Network Medicine, Brigham and Women's Hospital, Boston, MA, USA

**Keywords:** diet quality metrics, dietary diversity, nutrient adequacy, noncommunicable diseases, double burden of malnutrition, nutrition transition, nutritional epidemiology, India, South Asia, GDQS

## Abstract

**Background:**

In India, there is a need to monitor population-level trends in changes in diet quality in relation to both undernutrition and noncommunicable diseases.

**Objectives:**

We conducted a study to validate a novel diet quality score in southern India.

**Methods:**

We included data from 3041 nonpregnant women of reproductive age (15–49 years) from 2 studies in India. Diet was assessed using a validated food frequency questionnaire (FFQ). The Global Diet Quality Score (GDQS) was calculated from 25 food groups (16 healthy; 9 unhealthy), with points for each group based on the frequency and quantity of items consumed in each group. We used Spearman correlations to examine correlations between the GDQS and several nutrient intakes of concern. We examined associations between the GDQS [overall, healthy (GDQS+), and unhealthy (GDQS−) submetrics] and overall nutrient adequacy, micro- and macronutrients, body mass index (BMI), midupper arm circumference, hemoglobin, blood pressure, high density lipoprotein (HDL), and total cholesterol (TC).

**Results:**

The mean GDQS was 23 points (SD, 3.6; maximum, 46.5). In energy-adjusted models, positive associations were found between the overall GDQS and GDQS+ and intakes of calcium, fiber, folate, iron, monounsaturated fatty acid (MUFA), protein, polyunsaturated fatty acid (PUFA), saturated fatty acid (SFA), total fat, and zinc (ρ = 0.12–0.39; *P* < 0.001). Quintile analyses showed that the GDQS was associated with better nutrient adequacy. At the same time, the GDQS was associated with higher TC, lower HDL, and higher BMI. We found no associations between the GDQS and hypertension.

**Conclusions:**

The GDQS was a useful tool for reflecting overall nutrient adequacy and some lipid measures. Future studies are needed to refine the GDQS for populations who consume large amounts of unhealthy foods, like refined grains, along with healthy foods included in the GDQS.

## Introduction

In recent decades, India has witnessed an exponential increase in the burden of cardiometabolic diseases, with cardiovascular diseases contributing to 28.1% of total deaths and 14.1% of the total disability-adjusted life years in 2016 (1). The number of individuals with diabetes also increased substantially, from 26 million in 1990 to 65 million in 2016 (2). The latest National Family Health Survey–5 from 2019–2020 showed that in the southern state of Telangana, over 30% of men and women were overweight or obese (3). In contrast, 1 in 5 women of reproductive age (WRA) from rural areas in Telangana were undernourished, and nearly 60% of nonpregnant (NP) women of reproductive age in rural areas were found to be anemic (3). The coexistence of noncommunicable diseases (NCDs) alongside undernutrition presents a dire public health crisis in low- and middle-income countries (LMIC) like India and signifies a major challenge for sustainable human development in the 21^st^ century (4). There remains an urgent need to develop multidimensional interventional and policy approaches to deal with the unique challenges posed by the double burden of malnutrition.

Diet plays an important role for the prevention of both undernutrition and chronic diseases. The 2030 agenda for the Sustainable Development Goals recognizes nutrition as being crucial for ending hunger, achieving food security, and improving nutrition globally (5). In 2016, the WHO and FAO adopted the Rome Declaration on Nutrition which reaffirmed the need for more nutrition data and indicators for effective nutrition surveillance and policy-making (6). In particular, it was recognized that special attention should be given to nutritional issues for WRA (6). Although several global metrics of diet quality for WRA exist, such as the Minimum Dietary Diversity for Women (MDD-W) and the Prime Diet Quality Score, currently none can capture the double burden of malnutrition (7, 8).

Because the double burden of malnutrition is prevalent in resource-limited settings like India, it is imperative that dietary metrics to assess this be simple, cost-effective, and easy to administer. This paper presents results for an Indian setting from the development and validation of the Global Diet Quality Score (GDQS), a novel and simple food-based scoring system that aims to measure diet quality in relation to the double burden of malnutrition. The development of the GDQS adds to previous metrics like the MDD-W (7) by trying to measure diet quality in populations experiencing the double burden of malnutrition. This study examined associations of the GDQS with various NCD and undernutrition indicators among NP WRA—whose dietary habits and health status substantially influence maternal and children's health—from southern India, using data from the Andhra Pradesh Children and Parents Study (APCAPS) and the Indian Migration Study (IMS).

## Methods

### Study design and population

The study population included NP WRA who participated in the APCAPS and the IMS. The APCAPS was conducted in a rural/peri-urban population located near Hyderabad, the capital and the largest city of the Telangana state in southern India. Details about the APCAPS cohort have been previously published (9). For the current study, we included NP WRA who were part of the second (2009–2010) and third (2010–2012) waves of data collection. Participants who partook in multiple phases of the APCAPS were filtered to include data from only the earliest date of valid measurements to avoid overrepresentation of any single individual.

The IMS was established in 2005–2007 to investigate the effects of rural to urban migration on chronic disease risks in India among 6510 participants. The study used a sibling pair design to compare chronic disease risk factors in migrant urban factory workers and their spouses with those of their rural siblings. The IMS did not collect information on pregnancy status; however, some participants took part in the Hyderabad DXA Study (10), which required all women to be NP, and these women were included in the current analyses. All IMS participants who were included in the current analyses were from the Hyderabad area and attended the same clinics at the same time as the second wave of data collection for the APCAPS. Details of the IMS have been published elsewhere (11).

In this study, we excluded participants who were missing information on any of the diet quality scores, age, sex, or pregnancy status. Only NP women aged 15–49 years were included. Each variable was checked for outliers, and extreme values were removed (e.g., hemoglobin measurements >50 g/dl and plasma LDL concentrations >1000 mg/dL).

The APCAPS and IMS received approvals from the ethics committees of the National Institute of Nutrition, the Indian Council of Medical Research, and the London School of Hygiene and Tropical Medicine. Approval was also sought from the village heads and their committees in the study villages. The participants provided written informed consent, or a witnessed thumbprint if illiterate. The current study was approved by the Institutional Review Board at the Harvard T.H. Chan School of Public Health, Boston, MA.

### Exposure assessment

#### Dietary assessment

Diet was assessed by an interviewer-administered, validated, semi-quantitative FFQ that was developed for use in urban and rural India (12). In the prior FFQ validation study, the FFQ was validated against three 24-hour recalls among 530 factory workers and rural dwellers, with deattenuated Spearman correlations (*ρ*) ranging from 0.57 for total fat to 0.80 for protein. Food items that accounted for 90% of between-person variability and 90% of contributions to individual nutrient intakes were included in the FFQ. Study participants were asked to report the number of servings (e.g., bowl, ladle, raw number) and the unit of frequency (e.g., per day, per week, per month, per year) of 98 commonly consumed food items over the past year. Mixed dishes were disaggregated into individual ingredients based on the recipes that were specific to the Hyderabad region, which were provided by the National Institute of Nutrition, Hyderabad, India (unpublished work). Participants recorded the frequency of use of different types of cooking oil, and the most frequently used oil type was used in all recipe calculations for everyone. Where this information was unavailable, it was assumed that the individual used the same oil type as another member of the household if this information was present. This was necessary to distinguish between liquid and nonliquid cooking oils.

We calculated nutrient and food group intakes using nutrient databases developed in the IMS. Nutrient values for each ingredient were derived from the Indian food composition tables (13). For food items where data were not available, the USDA nutrient database (Release No.14) or McCance and Widdowsons Composition of Foods was used for nutrient composition (14, 15).

### Diet quality metrics

#### Global diet quality score

The GDQS is a global measure of diet quality (16) and is based entirely on 25 food groups. The GDQS food groups were adapted to represent foods in the Indian context. For example, due to high sugar contents in juice sold in India, we assumed that the items labeled as juice in the FFQ were actually referring to sugar-sweetened beverages. We did not have information from the FFQ for 4 categories of food groups [juice (categorized to sugar-sweetened beverages), processed meat, low fat dairy (2% or less fat content), and deep orange tubers], as these foods were not commonly consumed by APCAPS or IMS participants. Therefore, we assumed no consumption for these categories. For organ meats, based upon common recipes provided by the National Institute of Nutrition, it was assumed that all liver was chicken liver and all brain was lamb brain. Consequently, liver was added to the poultry category, while brain was added to the red meat category. Daily averages for each ingredient were determined, and each ingredient was linked to its corresponding food group. For each food group, a set of scores were computed by summing the daily ingredient amounts for all ingredients within a given food group and categorizing the intake levels to low, moderate, or high based on the predefined cutoff levels (16). The GDQS values in this cohort ranged from 0 to 46.5 points. We additionally computed the GDQS+ and GDQS− submetrics by only including 16 and 9 food groups, respectively. The GDQS+ values ranged from 0–29.5, while the GDQS− values ranged from 4–13 points.

#### MDD-W

The MDD-W was developed to measure nutrient intake adequacy among women living in under-resourced settings and in developing countries (7). The MDD-W is based on 10 food groups, including starchy staples, pulses, nuts and seeds, dairy, meat, poultry and fish, eggs, dark green leafy vegetables, other vitamin A–rich fruits and vegetables, other vegetables, and other fruits. The MDD-W scores range from 0 to 10, with 1 point allocated for each of the 10 food groups consumed over the last 24 hours and 0 points allocated otherwise.

#### Alternative Healthy Eating Index–2010

The Alternative Healthy Eating Index–2010 (AHEI-2010) is based on foods and nutrients that are predictive of chronic disease risk (17). For each of the 11 components, points range from a scale of 0 (poorest) to 10 (highest). Higher intakes of vegetables (excluding potatoes), fruit (excluding fruit juices), whole grains, nuts and legumes, long-chain n-3 fats, and PUFAs are scored positively, as is moderate alcohol consumption. In contrast, higher intakes of sugar-sweetened beverages and fruit juice, red and processed meats, trans fat, and sodium are scored in reverse. The AHEI-2010 scores range from 0 to 110, with higher scores indicating higher diet quality. We did not include information on alcohol and trans fat intakes for AHEI-2010 calculations, as the consumption information was not fully available.

### Outcome assessment

#### Nutrient measures

We calculated intakes of protein, fat, calcium, fiber, iron, cholesterol, zinc, vitamin A, folate, and vitamin B12 using nutrient composition tables from the Indian Food Composition Database (13). The nutrient adequacy score is a sum of binary adequacy for 8 component nutrients (i.e., protein, calcium, zinc, vitamin A, folate, vitamin B12, fiber, and iron) in terms of daily consumption for the appropriate age group based upon National Institutes of Health's dietary reference intakes (i.e., <19 years old or 19–49 years old).

#### Anthropometric data

Weight was measured twice to the nearest 0.1 kg without shoes using a digital SECA machine. Standing height was also measured twice without shoes to the nearest 1 mm with a Leicester plastic stadiometer (Chasmors Ltd.). The average of 2 measurements was used in this analysis. BMI was calculated as weight (kg)/height (m)^2^. Waist circumference (in cm) and midupper arm circumference (in cm) were measured using a nonstretchable metallic tape twice, and the average of the 2 measurements was used in the analyses.

#### Vascular and laboratory measures

Blood pressure was measured with a validated oscillometric device at the right arm in the supine position using appropriate cuff sizes (Omron M5-I). Two measurements were taken and averaged for analyses. Fasting blood samples (at least 8 hours) were collected in appropriate vacutainers, transferred within 1–2 hours (in an icebox at 4–8°C), and processed within 4 hours of collection. Glucose, triglycerides (TGs), total cholesterol, and HDL cholesterol were estimated with an auto analyzer (ACE Clinical System; Schiapparelli Biosystems) using the recommended kits (Alfa Wasserman). LDL cholesterol was estimated from triglycerides, total cholesterol, and HDL cholesterol using the Friedewald formula (18).

### Statistical analysis

We included the following variables to capture the NCD risk: BMI, waist circumference, blood pressure, fasting glucose, triglycerides (TGs), and total, LDL, and HDL cholesterol. All variables were checked for outliers, and extreme values were removed (e.g., hemoglobin measurements > 50 g/dl and LDL cholesterol > 1000 mg/dl). Listwise deletion was performed, where those who were missing any variables that are required for each analysis were removed from that analysis. We computed the means and SDs of continuous predictors or the counts and relative percentages within levels of categorical predictors. Descriptive characteristics of the study population were stratified by age (i.e., 15–29 and 30–49 years of age). Additionally, we tabulated the proportion of individuals in each food group consumption category (i.e., low, medium, high, and very high) (16). The cutoffs for those categories were developed from the analyses of FFQs and 24-hour recall data from diverse settings (16). The cutoffs were based on their ability to provide a reasonably even distribution of categorical consumption ranges.

We computed energy-adjusted nutrient intakes using the residual method (19). Spearman correlations between diet-quality metrics (AHEI-2010, GDQS, GDQS+, GDQS−, and MDD-W) and unadjusted and energy-adjusted daily nutrient intakes were computed. Across quintiles of the GDQS, we computed age-adjusted least square means of anthropometric outcomes, nutrient adequacy scores, and clinical outcomes. We ran the same models adjusting for education levels, occupation types, and standard of living index scores, and found no differences in substantive findings, except for in the GDQS− and waist circumference (*P* values changed from 0.033 to 0.077). We chose to include more parsimonious models’ results in this manuscript. Each of these outcomes was considered in both continuous and binary forms using predefined cutoffs of clinical significance (16). Coefficients for each quintile and 95% CIs were computed. We additionally report the overall *P* value for a linear trend across categories of the metrics. For the GDQS−, tertiles were used in place of quintiles due to a limited spread of the data. The results of the quintile analyses for AHEI-2010 and MDD-W are included in the **Supplemental Table 1**. Analyses were performed for the full cohort (20, 21). We used R (version 4.0.2) for all analyses.

## Results

The mean age of the women (*n* = 3041) in the cohort was 32.4 ± 10.7 years. The mean BMI in the cohort was in the normal range, although older women (30–49 years old) had a higher BMI than younger women at ages 15–29 years (22.8 compared with 19.5 kg/m^2^; **Table 1**). On average, the consumption levels of fiber, zinc, and folate were higher among younger women, while older women had higher intakes of calcium, vitamin A, and vitamin B12. Interestingly, younger women had higher scores on the GDQS and the GDQS+ submetric. In comparison, older women had a higher score on the GDQS− submetric, indicating lower consumption levels of unhealthy foods.

**TABLE 1:** Characteristics of the study participants who were nonpregnant women of reproductive age (15–49 years old) in the Andhra Pradesh Children and Parents Study and Indian Migration Study^1^

	Overall cohort	Younger women (15–29 years old)	Older women (30–49 years old)	*P* value^2^ (older compared
	*n* = 3041	*n* = 1449	*n* = 1592	with younger)
Age, years	32.4 (10.7)	22.2 (3.7)	41.8 (4.8)	
Anthropometry				
Height (cm)	152.0 (5.7)	152.8 (5.7)	151.3 (5.6)	<0.001
Weight, kg	49.2 (10.7)	45.6 (9.0)	52.4 (11.0)	<0.001
BMI, kg/m^2^	21.3 (4.3)	19.5 (3.5)	22.8 (4.4)	<0.001
Dietary intake				
Energy, kcal/d	1975 (695)	2043 (705)	1913 (681)	<0.001
Protein, g/d	47.9 (18.2)	49.2 (18)	46.7 (18)	<0.001
Protein, % energy	9.7 (1.1)	9.6 (1.0)	9.7 (1.3)	0.011
Fat, g/d	42.8 (23.0)	44.5 (23.0)	41.2 (24.0)	<0.001
Fat, % energy	19.2 (5.9)	19.3 (5.7)	19.0 (6.1)	0.127
Calcium, mg/d	483 (280)	463 (242)	501 (309)	<0.001
Fiber, g/d	8.0 (4.6)	8.3 (4.5)	7.7 (4.6)	0.001
Iron, mg/d	10.6 (5.6)	10.6 (4.9)	10.5 (6.1)	0.841
Cholesterol, mg/d	129 (85.0)	131 (87.0)	128 (84.0)	0.454
Zinc, mg/d	7.6 (2.7)	7.8 (2.7)	7.4 (2.7)	<0.001
Vitamin A, μg/d	622 (626)	569 (484)	670 (728)	<0.001
Folate, μg/d	201 (92.0)	208 (89.0)	194 (94.0)	<0.001
Vitamin B12, μg/d	3.1 (5.6)	2.5 (4.9)	3.6 (6.1)	<0.001
Diet scores				
GDQS	23.0 (3.6)	23.2 (3.5)	22.8 (3.7)	0.006
GDQS+	11.8 (4.0)	12.1 (3.8)	11.5 (4.1)	<0.001
GDQS−	11.3 (1.4)	11.1 (1.4)	11.4 (1.4)	<0.001
AHEI-2010	26.9 (4.8)	27.1 (4.8)	26.7 (4.9)	0.010
MDD-W	6.3 (1.5)	6.3 (1.4)	6.3 (1.5)	0.223

1 Values are means (SDs) unless otherwise noted. AHEI-2010, Alternative Healthy Eating Index–2010; GDQS, Global Diet Quality Score; GDQS−, Global Diet Quality Score negative submetric; GDQS+, Global Diet Quality Score positive submetric; MDD-W, Minimum Diet Diversity for Women.

2 Welch 2-sample *t*-test.

In this study population, consumption of fruits, including citrus (83.9%) and deep orange fruits (89.1%), was low (**Table 2**). However, a larger percentage of women (77.7%) consumed medium to high amounts of other fruits, like apples, jackfruit, and tamarind. More than 90% of women had low consumption levels of cruciferous vegetables and deep orange vegetables, while more than half of the women consumed medium to high amounts of dark green leafy vegetables. Nearly 70% consumed whole grains in medium to high category levels of the GDQS, while all women consumed high amounts of refined grains and baked goods. Interestingly, more than 70% of the study population had medium to high consumption levels of plant protein in the form of legumes and nuts and seeds. The predominant form of animal protein that was consumed was eggs, with about 46% of women having medium to high consumption levels. In contrast, consumption levels of poultry, fish, and red meat were much lower, with 65.2% of women consuming low amounts of poultry, 93.2% consuming low amounts of fish, and nearly 81% consuming low amounts of red meat. Processed meat was not consumed in this cohort. While sugar-sweetened beverages were not widely consumed, nearly 80% of participants consumed medium to high amounts of high fat dairy. Likewise, the majority had considerably high consumption of liquid oils (94.9%).

**TABLE 2:** Distributions of the categories for Global Diet Quality Scores^1^

GDQS Submetric	Categories^2^	Low	Medium	High	Very high^3^
Positive	Citrus fruits	83.9	15.2	0.9	NA
	Cruciferous vegetables	95.3	4.7	0.1	NA
	Dark green leafy vegetables	43.1	54.1	2.9	NA
	Deep orange fruits	89.1	10.1	0.8	NA
	Deep orange vegetables	93.7	5.9	0.4	NA
	Deep orange tubers^4^	100	0.0	0.0	NA
	Eggs	54.0	41.9	4.1	NA
	Fish and shellfish	93.2	6.4	0.5	NA
	Whole grains	26.7	26.2	47.1	NA
	Low fat dairy^4^	100	0.0	0.0	NA
	Legumes	3.0	59.8	37.3	NA
	Liquid oils	4.0	1.1	94.9	NA
	Nuts and seeds	19.8	54.1	26.1	NA
	Other fruits	22.3	48.6	29.1	NA
	Other vegetables	0.9	72.3	26.7	NA
	Poultry and game meat	65.2	33.5	1.3	NA
Negative	Processed meat^4^	100	0.0	0.0	NA
	Red meat^5^	80.5	19.4	0.1	NA
	High fat dairy^6^	10.9	7.8	71.1	10.1
	Refined grains and baked goods	0.0	0.0	100	NA
	Juice^7^	100	0.0	0.0	NA
	Sugar-sweetened beverages	89.8	9.1	1.0	NA
	Sweets and ice cream	13.7	35.1	51.3	NA
	White roots and tubers	94.4	5.4	0.2	NA
	Purchased deep fried foods	28.4	54.4	17.3	NA

1 The values are in percentages. GDQS, Global Diet Quality Score; NA, not applicable.

2 The categorization of the intake levels was done based on the application of Nurses’ Health Study's FFQ standard portion sizes to Prime Diet Quality Score frequency groups. We doubled the cutoffs for refined grains and added a fourth scoring category for high fat dairy (equivalent to 3 + servings). Further adjustments were made to the cutoffs based on the analysis conducted for the operationalization paper included in this supplement (22).

3 The “very high” category only applied to the high fat dairy category.

4 Deep orange tubers, low fat dairy, and processed meat consumption is uncommon in this population; therefore, all were assumed to have low levels of consumption.

5 Red meat is nonlinearly scored (0, 1, 0 points).

6 Points for high fat dairy categories were nonlinear.

7 Due to high sugar contents in juice sold in India, we categorized all juice consumption under sugar-sweetened beverages.

The mean GDQS value was 23.0 (SD, 3.6), with a possible maximum point of 49 points. While GDQS+ values were roughly normally distributed, with a mean score of 11.8 (SD, 4.0), the distribution of the GDQS− values was slightly left-skewed, with a mean score of 11.3 (median, 11.0; **Supplemental Figure 1**). **Table 3** shows the correlations between each score and the energy-adjusted nutrient intake estimated from the FFQ, as well as other scores. The GDQS was more correlated with intakes of folate (*ρ =* 0.35), fiber (*ρ =* 0.29), total fat (*ρ =* 0.26), iron (*ρ =* 0.25), zinc (*ρ =* 0.23), and protein (*ρ =* 0.23) than other nutrients. The GDQS+ was positively correlated with intakes of folate (*ρ =* 0.39), total fat (*ρ =* 0.34), fiber (*ρ =* 0.31), and iron (*ρ =* 0.27) and inversely correlated with intake of vitamin B12 (*ρ =* −0.14). As expected, the GDQS− submetric was inversely associated with intakes of total fat (*ρ =* −0.31), saturated fat (*ρ =* −0.23), and calcium (*ρ =* −0.23). The AHEI-2010 was positively correlated with intakes of polyunsaturated fat (*ρ =* 0.62), total fat (*ρ =* 0.39), and iron (*ρ =* 0.30), while the MDD-W was positively correlated with intakes of folate (*ρ =* 0.37), protein (*ρ =* 0.35), and total fat (*ρ =* 0.34). The GDQS was very strongly correlated with the GDQS+ (*ρ =* 0.94) and the MDD-W (*ρ =* 0.72) and was not correlated with the GDQS− (*ρ =* −0.08). The AHEI-2010 was most strongly correlated with the GDQS+ submetric (*ρ =* 0.43).

**TABLE 3:** Correlation between diet quality scores and estimated nutrient intake^1^

	GDQS		GDQS+		GDQS−		AHEI-2010		MDD-W	
	ρ	*P*	ρ	*P*	ρ	*P*	ρ	*P*	ρ	*P*
Nutrient intake										
Calcium	0.10	<0.001	0.18	<0.001	−0.23	<0.001	0.19	<0.001	0.24	<0.001
Fiber	0.29	<0.001	0.31	<0.001	−0.12	<0.001	0.25	<0.001	0.23	<0.001
Folate	0.35	<0.001	0.39	<0.001	−0.21	<0.001	0.30	<0.001	0.37	<0.001
Iron	0.25	<0.001	0.27	<0.001	−0.11	<0.001	0.32	<0.001	0.27	<0.001
Monounsaturated fat	0.21	<0.001	0.25	<0.001	−0.18	<0.001	0.03	0.119	0.26	<0.001
Protein	0.23	<0.001	0.25	<0.001	−0.12	<0.001	0.17	<0.001	0.35	<0.001
Polyunsaturated fat	0.20	<0.001	0.24	<0.001	−0.17	<0.001	0.62	<0.001	0.19	<0.001
Saturated fat	0.10	<0.001	0.17	<0.001	−0.23	<0.001	0.03	0.108	0.23	<0.001
Total fat	0.26	<0.001	0.34	<0.001	−0.31	<0.001	0.39	<0.001	0.34	<0.001
Vitamin A	0.14	<0.001	0.12	<0.001	0.01	0.414	0.12	<0.001	0.18	<0.001
Vitamin B12	−0.11	<0.001	−0.14	<0.001	0.11	<0.001	0.01	0.502	−0.03	0.147
Zinc	0.23	<0.001	0.20	<0.001	0.03	0.156	0.05	0.008	0.26	<0.001
Score										
GDQS	1		0.94	<0.001	−0.08	<0.001	0.38	<0.001	0.72	<0.001
GDQS+	—		1		−0.39	<0.001	0.43	<0.001	0.76	<0.001
GDQS−	—		—		1		−0.24	<0.001	−0.29	<0.001
AHEI-2010	—		—		—		1		0.34	<0.001
MDD-W	—		—		—		—		1	

1 All estimated nutrient intakes were adjusted for energy. *P* values for score correlations are based upon asymptotic approximations of the t/F distribution. AHEI-2010, Alternative Healthy Eating Index–2010; GDQS, Global Diet Quality Score; GDQS−, Global Diet Quality Score negative submetric; GDQS+, Global Diet Quality Score positive submetric; MDD-W, Minimum Diet Quality for Women.

In the quintile regression with continuous outcomes (**Table 4**), BMI, midupper arm circumference, and waist circumference were higher with higher values of the GDQS and GDQS+. As expected, the nutrient adequacy score was higher with higher scores on the GDQS and GDQS+ submetric. However, with higher GDQS and GDQS+ values, total cholesterol was higher while HDL cholesterol was lower. No associations were seen between the GDQS and GDQS+ values and hemoglobin, fasting glucose, blood pressure measures, and triglyceride concentrations. These trends tended to differ with the GDQS−. Unlike the GDQS and GDQS+, increasing values of the GDQS− were associated with lower BMI, midupper arm circumference, and waist circumference values. Most notably, increasing values of the GDQS− were associated with lower nutrient adequacy scores. We found no associations between the GDQS− and measures of hemoglobin, fasting glucose, blood pressure, or lipids.

**TABLE 4:** Quintile or tertile analyses of Global Diet Quality Scores against continuous values of nutritional status and noncommunicable disease indicators^1^

	Outcome						
Outcomes category	Groups	G1	G2	G3	G4	G5	*P* value, linear trend^2^
GDQS	*n*	831	608	570	543	—	
Anthropometry	BMI, kg/m^2^	20.5 (20.3–20.8)	21.0 (20.7–21.3)	21.2 (20.9–21.5)	21.6 (21.3–22.0)	22.4 (22.1–22.8)	<0.001
	Midupper arm circumference, cm	24.1 (23.9–24.3)	24.5 (24.2–24.7)	24.8 (24.6–25.1)	25.0 (24.7–25.3)	25.5 (25.2–25.8)	<0.001
	Waist circumference, cm	68.1 (67.4–68.7)	69.6 (68.8–70.4)	70.2 (69.4–71.0)	71.2 (70.3–72.1)	73.2 (72.4–74.0)	<0.001
Nutrient/nutrient biomarker	Nutrient adequacy score (0–8)^3^	3.63 (3.56–3.69)	4.09 (4.02–4.16)	4.38 (4.31–4.46)	4.54 (4.46–4.62)	4.28 (4.20–4.35)	<0.001
	Hemoglobin, g/dl	11.9 (11.8–12.0)	12.0 (11.9–12.1)	12.0 (11.8–12.1)	11.9 (11.8– 12.1)	12.0 (11.9–12.2)	0.613
	Glucose, mg/dL	91.4 (90.1–92.8)	92.5 (90.9–94.1)	91.2 (89.6–92.9)	91.1 (89.2–92.9)	93.4 (91.7–95.1)	0.337
BP	Systolic BP, mmHg	115 (114–116)	115 (114–116)	115 (114–116)	115 (114–116)	115 (114–116)	0.924
	Diastolic BP, mmHg	75.0 (74.3–75.7)	75.5 (74.6–76.3)	75.8 (74.9–76.6)	75.7 (74.8–76.6)	75.2 (74.3–76.1)	0.663
Lipid	Total cholesterol, mg/dL	156 (153–159)	157 (153–160)	158 (155–162)	161 (157–165)	165 (162–169)	0.000
	HDL cholesterol, mg/dL	45.0 (44.2–45.8)	44.1 (43.2–45.0)	44.3 (43.4–45.3)	42.9 (41.9–43.9)	42.8 (41.8–43.7)	<0.001
	Triglycerides, mg/dL	109 (105–113)	107 (102–112)	107 (102–112)	113 (108–119)	107 (101–112)	0.804
	Groups	G1	G2	G3	G4	G5	
GDQS+	*n*	708	558	591	598	576	
Anthropometry	BMI, kg/m^2^	20.5 (20.2–20.8)	20.8 (20.5–21.2)	21.0 (20.7–21.4)	21.7(21.4–22.0)	22.5 (22.2–22.8)	<0.001
	Midupper arm circumference, cm	24.0 (23.8–24.3)	24.3 (24.0–24.6)	24.6 (24.3–24.9)	25.0 (24.8–25.3)	25.6 (25.4–25.9)	<0.001
	Waist circumference, cm	68.1 (67.3–68.8)	68.8 (67.9–69.6)	69.8 (69.0–70.6)	71.2 (70.4–71.9)	73.6 (72.8–74.4)	<0.001
Nutrient/nutrient biomarker	Nutrient adequacy score (0–8)^3^	3.49 (3.43–3.56)	4.05 (3.97–4.12)	4.37 (4.30–4.44)	4.55 (4.48–4.62)	4.27 (4.20–4.34)	<0.001
	Hemoglobin, g/dl	11.9 (11.8–12.0)	12.0 (11.9–12.2)	12.0 (11.9–12.1)	12.0 (11.8–12.1)	11.9 (11.8–12.1)	0.88
	Glucose, mg/dL	91.5 (90.0–93.0)	92.4 (90.8–94.1)	91.3 (89.6–92.9)	91.5 (89.9–93.2)	92.9 (91.2–94.5)	0.48
BP	Systolic BP, mmHg	115 (114–116)	115 (114–116)	115 (114–116)	116 (115–117)	115 (114–116)	0.68
	Diastolic BP, mmHg	75.1 (74.3–75.9)	75.2 (74.4–76.1)	75.4 (74.6–76.2)	75.9 (75.1–76.7)	73.3 (74.5–76.2)	0.38
Lipid	Total cholesterol, mg/dL	154 (150–157)	156 (152–159)	158 (154–161)	162 (159–165)	167 (163–170)	<0.001
	HDL cholesterol, mg/dL	44.9 (44.1–45.8)	44.5 (43.6–45.5)	44.1 (43.1–45.0)	43.6 (42.7–44.5)	42.5 (41.6–43.5)	<0.001
	Triglycerides, mg/dL	110 (105–114)	107 (102–112)	108 (103–113)	112 (107–117)	106 (101–111)	0.821
	Groups	G1	G2	G3	—	—	
GDQS−^4^	*n*	1540	999	490	—	—	
Anthropometry	BMI, kg/m^2^	21.6 (21.4–21.8)	21.0 (20.8–21.3)	20.7 (20.4–21.1)	—	—	0.373
	Midupper arm circumference, cm	25.0 (24.8–25.1)	24.4 (24.2–24.7)	24.3 (24.0–24.6)	—	—	0.031
	Waist circumference, cm	70.9 (70.4–71.4)	69.3 (68.7–69.9)	69.7 (68.8–70.6)	—	—	0.033
Nutrient/nutrient biomarker	Nutrient adequacy score (0–8)^3^	4.23 (4.18–4.28)	4.04 (3.98–4.10)	3.96 (3.88–4.04)	—	—	<0.001
	Hemoglobin, g/dl	11.9 (11.8–12.0)	12.1 (12.0–12.2)	11.9 (11.7–12.1)	—	—	0.689
	Glucose, mg/dL	91.4 (90.4–92.4)	92.4 (91.2–93.7)	92.5 (90.7–94.3)	—	—	0.168
BP	Systolic BP, mmHg	115 (115–116)	115 (114–116)	115 (113–116)	—	—	0.818
	Diastolic BP, mmHg	75.6 (75.0–76.1)	75.4 (74.7–76.0)	74.9 (74.0–75.8)	—	—	0.780
Lipid	Total cholesterol, mg/dL	162 (160–164)	157 (155–160)	153 (149–157)	—	—	0.818
	HDL cholesterol, mg/dL	43.7 (43.2–44.3)	44.1 (43.4–44.8)	44.4 (43.4–45.3)	—	—	0.361
	Triglycerides, mg/dL	108 (104–111)	110 (106–114)	108 (102–113)	—	—	0.784

1 All models are adjusted for age. Values are estimated marginal means (95% CIs). Groups 1–5 were quintile or tertile groups. BP, blood pressure; G, group; GDQS, Global Diet Quality Score; GDQS−, Global Diet Quality Score negative submetric; GDQS+, Global Diet Quality Score positive submetric.

2 The *t*-test for linear trend (Pr(>|t|)) tested whether a coefficient of a linear trend for the ordered factor is different than 0.

3 The nutrient adequacy score is a sum of binary adequacy for 8 component nutrients (i.e., protein, calcium, zinc, vitamin A, folate, vitamin B12, fiber, and iron) in terms of daily consumption for the appropriate age group based upon National Institutes of Health's dietary reference intakes (i.e., <19 years old or 19–49 years old).

4 GDQS− analyses are reported as tertiles due to a limited spread of the data.

## Discussion

In this cross-sectional analysis of an urbanizing South Indian population, we found that a global metric of diet quality was very strongly associated with measures of nutrient adequacy but less so with cardiometabolic outcomes. In particular, the GDQS and the GDQS+ submetric were positively associated with the macronutrients protein and fat and several micronutrients that are of nutritional concern in LMICs, such as folate, fiber, and iron. While the positive submetric was more strongly associated with these nutrients, the GDQS− submetric was inversely associated with saturated fat. Interestingly, although the GDQS and the GDQS+ submetric were adversely associated with anthropometric measures and lipid measures, higher scores on the GDQS− submetric were associated with lower BMI, midupper arm circumference, and waist circumference values.

It is notable that both the GDQS and GDQS+ were correlated with intakes of folate and iron, 2 micronutrients that are of particular concern among women of reproductive age in India (23, 24). Recent surveys estimate that close to 75% of Indian women are folate insufficient (23), while nearly half are iron-deficient (24). Most recently, WRA were included as beneficiaries under the National Iron Plus Initiative of the Government of India. Under this program, WRA receive 100 mg of elemental iron and 500 ug of folic acid weekly throughout the year (25). However, the uptake of this program and the coverage of beneficiaries has been poor at the national level (26). It is therefore especially significant that the GDQS was able to assess nutrient adequacy in this demographic group. Despite evidence of associations between the scores and intakes of iron and an overall measure of nutrient adequacy, it is also important to note that there was no clear evidence of an association between the GDQS, AHEI-2010, or MDD-W and hemoglobin concentrations in this population. This is an area that requires future research, as anemia is common among WRA in India, with over 50% of WRA estimated to be anemic in 2016 (27). The null association with hemoglobin concentrations may be due to the fact that over 90% of the total iron present in the Indian diet is nonheme iron, which has much lower bioavailability (28). Additionally, iron absorption inhibitors that are highly prevalent in the Indian diet present an India-specific challenge in preventing anemia. For example, black tea, which is a rich source of polyphenols and can inhibit iron absorption (29), was not included in the score calculation but is widely and frequently consumed. Legumes contain phytates, another potential inhibitor for iron absorption, are also consumed frequently in this population and are scored positively in the GDQS.

While the MDD-W showed similar associations with micronutrients, it is important to note that the GDQS was designed to capture the risks of both undernutrition and overnutrition, and may therefore be used in communities facing the double burden of malnutrition. Interestingly, in this population, higher scores on both the GDQS and GDQS+ were associated with higher anthropometric measures, including BMI, midupper arm circumference, and waist circumference values. However, it is worth mentioning that BMIs and waist circumferences were in the healthy range across quintiles of the GDQS metric. The higher BMIs with higher GDQS scores may simply reflect better food security and access to a wider variety of foods. On the contrary, we did find that higher scores on the GDQS− submetric, indicating less consumption of unhealthy foods, tended to be associated with a lower BMI and a lower waist circumference. Notably, foods that make up the GDQS− submetric, including red meat, processed meat, refined grains, sugar-sweetened beverages, sweets, and fried foods, have all been previously reported to be associated with adverse cardiometabolic outcomes (30–34). In a previous publication of the IMS data, an “animal food” dietary pattern characterized by intakes of red meat, poultry, fish/seafood, and eggs was adversely associated with cardiometabolic risk factors (35). Although a majority of participants consumed low amounts of red meat, sugar-sweetened beverages, and white roots and tubers, all participants consumed high amounts of refined grains and baked goods, and a significant proportion consumed fried foods. This pattern of distribution is reminiscent of the early stages of the nutrition transition (36) and may signal more adverse changes to the diet for this urbanizing community. Previous research from the subcontinent has shown that excessive consumption of red meat showed a stronger association with cardiovascular disease than the protective effects of physical activity (37).

There were several other findings that were unique to this study setting. Most notable was the evidence of a negative association of the GDQS with HDL cholesterol and a positive association with total cholesterol. These associations may be driven by dietary patterns of the subcontinent. For instance, legumes, termed as the poor man's rich protein, are often consumed with large amounts of white rice and other refined grain products. The most recent multinational Prospective Urban Rural Epidemiology study showed that higher intake of white rice (≥450 g/day compared with <150 g/day) was associated with a 20% higher risk of diabetes, with a nearly 60% higher risk among participants from South Asia (38). Another multi-ethnic study showed that carbohydrate intake may partially explain poor lipid profiles among South Asians (39). While it may not be feasible to convince the Indian population to replace white rice with brown rice (40), a possible behavioral change may be to decrease the proportion of white rice and increase the proportion of legumes. In fact, in 1 study of Costa Ricans that follow a staple dietary pattern of white rice and beans, a higher proportion of beans to rice (2:1 and 3:1) was associated with higher HDL cholesterol, lower concentrations of triglycerides and fasting glucose, and lower odds of metabolic syndrome (41). Of note, there were several food items that were commonly consumed in this population (e.g., coconuts) but were not included in this validation study, as they did not fit into the categories chosen during the development of the GDQS. Future work can modify the GDQS to fit culturally specific foods that may be associated with nutrient adequacy and disease risks.

Previous research has shown that South Asians suffer a disproportionately higher burden of cardiometabolic diseases compared to non-Hispanic whites and other Asian groups (42, 43). These findings on South Asian immigrants in Western countries serve as an example of the potential health impacts of dietary shifts as India goes through a period of unprecedented socioeconomic development. In this context, the GDQS provides a flexible framework for adjusting for dietary shifts and capturing diet quality as the country undergoes nutrition transition.

Our study has several strengths. The availability of a previously validated, ethnic-specific FFQ to measure diets allowed us to capture foods that are unique to this region. In addition, the use of a well-phenotyped study population that lives in an urbanizing community allowed us to examine associations between diet and various cardiometabolic risk factors. However, there are several limitations that need to be recognized. First, our study is cross-sectional in nature, thereby limiting us to make causal inferences of observed associations. Second, while the GDQS was intended for NP and nonlactating women of reproductive age, our data set did not have information on lactation status.

In conclusion, the GDQS is a promising and simple-to-use tool to monitor population-level changes in diet quality, especially for undernutrition-related indicators, in India. While the GDQS offers a simple tool to monitor changes in diet quality in urbanizing areas of India, there are some context-specific challenges. Further refinement of the scores for the Indian context are needed. Future research should also focus on understanding how longitudinal changes in the GDQS influence subsequent disease risks.

## Supplementary Material

nxab217_Supplemental_File

## Notes

Funding was provided by FHI Solutions, recipient of a Bill & Melinda Gates Foundation grant to support Intake–Center for Dietary Assessment. Andhra Pradesh Children and Parents Study data collection was funded by the Wellcome Trust (grant numbers 083707, 084774, and 084754) and the Indian Migration Study data collection was funded by the Wellcome Trust (grant number 070797).

Author disclosures: TTF is an Associate Editor for the *Journal of Nutrition* and played no role in the *Journal*’s evaluation of the manuscript. All other authors report no conflicts of interest.

Supplemental Figure 1 and Supplemental Table 1 are available from the “Supplementary data” link in the online posting of the article and from the same link in the online table of contents at https://academic.oup.com/jn/.

Published in a supplement to the Journal of Nutrition. Publication costs for this supplement were funded by the Bill & Melinda Gates Foundation in conjunction with FHI Solutions, recipient of a Bill & Melinda Gates Foundation grant to support Intake–Center for Dietary Assessment. The opinions expressed in this publication are those of the authors and are not attributable to the sponsors or the publisher, Editor, or Editorial Board of the *Journal of Nutrition*. The Supplement Coordinators for the supplement publication were Megan Deitchler, Intake–Center for Dietary Assessment at FHI Solutions, Washington, DC; and Sabri Bromage, Harvard T.H. Chan School of Public Health, Boston, MA.

The GDQS research initiative was launched by Intake – Center for Dietary Assessment. The research was led by Harvard T.H. Chan School of Public Health, Department of Nutrition and carried out in collaboration with researchers at the National Public Health Institute (INSP), Mexico. Funding for the research was provided by FHI Solutions, recipient of a Bill & Melinda Gates Foundation grant to support Intake – Center for Dietary Assessment.

Abbreviations used: AHEI-2010, Alternative Healthy Eating Index–2010; APCAPS, Andhra Pradesh Children and Parents Study; FFQ, Food Frequency Questionnaire; GDQS, Global Diet Quality Score; GDQS−, Global Diet Quality Score negative submetric; GDQS+, Global Diet Quality Score positive submetric; IMS, India Migration Study; LMIC, low- and middle-income countries; MDD-W, Minimum Dietary Diversity for Women; NCD, noncommunicable diseases; NP, nonpregnant; WRA, women of reproductive age.
