# Supplementary material for: Validation of Global Diet Quality Score Among Nonpregnant Women of Reproductive Age in India: Findings from the Andhra Pradesh Children and Parents Study (APCAPS) and the Indian Migration Study (IMS)
Source: J Nutr. 2021 Oct 23;151(Suppl 2):101S–109S. doi: 10.1093/jn/nxab217 (PMC8564710; doi:10.1093/jn/nxab217)
Supplement: nxab217_Supplemental_File [file nxab217_Supplemental_File.docx]

**Validation of Global Diet Quality Score among non-pregnant women of reproductive age in India: Findings from the Andhra Pradesh Children and Parents Study (APCAPS) and the Indian Migration Study (IMS)**

Mika Matsuzaki

Online Supplementary Material

**Supplemental figure 1: Distribution of Global Diet Quality Scores (overall, positive, and negative) among non-pregnant women of reproductive age (n = 3041)**


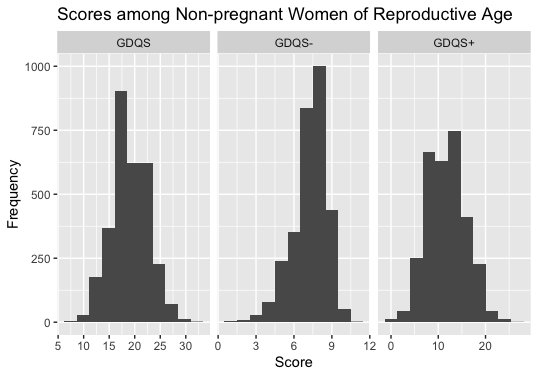


**Supplemental Table 1: Quintile analyses Minimum Diet Diversity for Women (MDD-W) and Alternate Healthy Eating Index 2010 (AHEI-2010) against continuous values of nutritional status and non-communicable disease indicators.**

| **Outcomes category** | **Outcome** |  | **Q1** | **Q2** | **Q3** | **Q4** | **Q5** | **P-value, linear trend** |
| --- | --- | --- | --- | --- | --- | --- | --- | --- |
| AHEI-2010 |  | n**^1^** | 892 | 824 | 674 | 414 | 213 |  |
| Anthropometry | BMI (kg/m^2^) | 2952 | 20.4(20.1 to 20.7) | 20.6(20.4 to 20.9) | 21.4(21.0 to 21.7) | 21.9(21.6 to 22.3) | 22.6(22.3 to 23.0) | <0.001 |
|  | Mid-upper arm circumference (cm) | 3021 | 24.1(23.9 to 24.4) | 24.2(23.9 to 24.4) | 24.7(24.4 to 25.0) | 25.2(24.9 to 25.4) | 25.8(25.5 to 26.0) | <0.001 |
|  | Waist circumference (cm) | 3026 | 68.1(67.4 to 68.8) | 68.8(68.1 to 69.5) | 70.3(69.5 to 71.2) | 71.5(70.7 to 72.3) | 73.8(72.9 to 74.6) | <0.001 |
| Nutrient/nutrient biomarker | Nutrient adequacy score (0-8) | 3032 | 3.89(3.82 to 3.96) | 4.14(4.08 to 4.21) | 4.19(4.1 to 4.27) | 4.28(4.2 to 4.36) | 4.19(4.11 to 4.27) | <0.001 |
|  | Hemoglobin (g/dL) | 3032 | 12.0(11.8 to 12.1) | 12.0(11.9 to 12.2) | 11.9(11.7 to 12.1) | 12.0(11.8 to 12.1) | 12.0(11.8 to 12.1) | 0.939 |
|  | Glucose (mg/dL) | 2949 | 91.5(90.1 to 93.0) | 92.4(90.9 to 93.8) | 92.0(90.2 to 93.8) | 92.5(90.8 to 94.1) | 91.0(89.3 to 92.8) | 0.727 |
| BP | Systolic blood pressure (mmHg) | 3030 | 115(114 to 116) | 115(114 to 116) | 115(113 to 116) | 115(114 to 116) | 116(115 to 117) | 0.063 |
|  | Diastolic blood pressure (mmHg) | 3030 | 75.2(74.4 to 75.9) | 75.4(74.6 to 76.1) | 74.8(73.9 to 75.7) | 75.7(74.9 to 76.6) | 76.1(75.2 to 77.0) | 0.091 |
| Lipid | Total cholesterol (mg/dL) | 2947 | 158(155 to 162) | 156(153 to 159) | 157(153 to 161) | 162(159 to 166) | 162(158 to 166) | 0.016 |
|  | HDL cholesterol (mg/dL) | 3032 | 44.6(43.7 to 45.4) | 44.5(43.6 to 45.3) | 43.4(42.4 to 44.4) | 44.6(43.6 to 45.6) | 42.2(41.2 to 43.2) | 0.002 |
|  | Triglycerides (mg/dL) | 2939 | 111(107 to 116) | 108(104 to 113) | 106(101 to 112) | 107(102 to 113) | 108(102 to 113) | 0.335 |
| **MDD-W** |  | **n^1^** | **738** | **740** | **486** | **553** | **514** |  |
| Anthropometry | BMI (kg/m^2^) | 2952 | 20.5(20.2 to 20.8) | 20.9(20.7 to 21.2) | 21.5(21.2 to 21.8) | 22.4(22.0 to 22.8) | 22.9(22.4 to 23.5) | <0.001 |
|  | Mid-upper arm circumference (cm) | 3021 | 24.1(23.9 to 24.4) | 24.4(24.2 to 24.6) | 24.9(24.6 to 25.1) | 25.6(25.3 to 25.9) | 25.9(25.5 to 26.4) | <0.001 |
|  | Waist circumference (cm) | 3026 | 68.0(67.4 to 68.7) | 69.4(68.7 to 70.0) | 70.8(70.0 to 71.5) | 73.4(72.5 to 74.3) | 74.5(73.2 to 75.8) | <0.001 |
| Nutrient/nutrient biomarker | Nutrient adequacy score (0-8) | 3032 | 3.6(3.54 to 3.66) | 4.26(4.2 to 4.32) | 4.45(4.39 to 4.52) | 4.44(4.36 to 4.53) | 4.19(4.07 to 4.31) | <0.001 |
|  | Hemoglobin (g/dL) | 3032 | 11.9(11.8 to 12.0) | 11.9(11.8 to 12.1) | 11.9(11.8 to 12.1) | 12.1(11.9 to 12.2) | 12.0(11.8 to 12.3) | 0.243 |
|  | Glucose (mg/dL) | 2949 | 91.5(90.2 to 92.8) | 92.0(90.7 to 93.4) | 92.2(90.7 to 93.7) | 91.2(89.3 to 93.1) | 92.8(90.2 to 95.5) | 0.577 |
| BP | Systolic blood pressure (mmHg) | 3030 | 114(114 to 115) | 115(113.8 to 116) | 116(115 to 117) | 116(115 to 117.) | 115(113 to 116) | 0.453 |
|  | Diastolic blood pressure (mmHg) | 3030 | 74.9(74.3 to 75.6) | 75.1(74.4 to 75.8) | 75.7(74.9 to 76.5) | 76.6(75.6 to 77.6) | 75.1(73.8 to 76.5) | 0.251 |
| Lipid | Total cholesterol (mg/dL) | 2947 | 152(149 to 154) | 155(152 to 158) | 163(160 to 166) | 169(164 to 173) | 171(165 to 176) | <0.001 |
|  | HDL cholesterol (mg/dL) | 3032 | 44.8(44.1 to 45.6) | 44.3(43.5 to 45.1) | 43.6(42.8 to 44.5) | 43.2(42.1 to 44.4) | 41.6(40.0 to 43.1) | <0.001 |
|  | Triglycerides (mg/dL) | 2939 | 106(102 to 110) | 107(102 to 111) | 113(108 to 118) | 111(105 to 117) | 104(96.0 to 113) | 0.928 |

1. n for each quintile is based on all the women of reproductive age included in the current manuscript, some of which were missing the indicators included in the analyses.
